# Research on related factors of hyperuricemia in high altitude area migrant population

**DOI:** 10.3389/fendo.2025.1559260

**Published:** 2025-07-07

**Authors:** Dong-Dong Meng, Yin-Dong Kang, De-Hui Chang

**Affiliations:** Department of Urology, 940 Hospital of the Joint Service Support Force of the Chinese People’s Liberation Army, Lanzhou, Gansu, China

**Keywords:** high altitude area, hyperuricemia, multivariate logistic regression, creatinine, red blood cell count, plateau migrants

## Abstract

**Background:**

Hyperuricemia, a prevalent metabolic disorder, is witnessing a global annual increase in incidence. The gout it triggers and its link to other chronic diseases pose a severe threat to human health. The unique natural environment of high-altitude regions, characterized by low oxygen partial pressure and cold climate, may exert a distinctive influence on human metabolism, thereby impacting the onset and progression of hyperuricemia.

**Methods:**

This study recruited 284 plateau migrants undergoing physical examinations at Ritu County Hospital from June to December 2024. Among them, 224 had hyperuricemia and 60 had normal uric acid levels. It collected various indicators of all subjects, including general demographic information, blood routine parameters, and biochemical markers. For univariate analysis, the t-test was used for continuous variables and the chi-square test for categorical variables to screen potential factors related to hyperuricemia. Then, a multicollinearity analysis was done on the univariate factors. After excluding variables with a VIF greater than 5, the remaining ones were put into the multivariate logistic regression model to identify the independently related factors of hyperuricemia.

**Result:**

The incidence of hyperuricemia in the study population was 78.87%. Variables such as gender, age, red blood cell count and creatinine were found to be independently associated with hyperuricemia.

**Conclusion:**

This study revealed an elevated incidence of hyperuricemia in high-altitude area migrants and identified its independent related factors, offering a crucial foundation for the prevention and treatment of hyperuricemia in these regions.

## 1 Introduction

Hyperuricemia, a common metabolic disorder, has been experiencing a rising prevalence worldwide year by year (1), drawing extensive public health attention. According to relevant statistics, the prevalence of hyperuricemia in the general population can reach approximately 10% - 20% (2, 3), and in certain specific regions or populations, this proportion may be even higher (4). In high-altitude areas, the unique geographical environment and climatic conditions render the prevalence of this disease more complex. Data from the Tibet Autonomous Region Center for Disease Control and Prevention show that the prevalence of hyperuricemia among residents aged ≥18 years in Tibet is 16.86%, far higher than the national average in China (5). Plateau migrants exhibit more severe hyperuricemia due to their bodies’ maladaptation to hypoxic environments (6, 7).

Previous research has indicated that factors such as low oxygen partial pressure, low temperature, and intense ultraviolet radiation in high altitude environments may disrupt the normal metabolic processes of the human body (8, 9), potentially affecting uric acid metabolism in migrants (10–12). Studies have found that individuals traveling to high-altitude areas, acutely exposed to high altitude, or long-term residents of high-altitude regions exhibit elevated uric acid levels in serum or urine (13), and there is a significant correlation between uric acid levels and red blood cell count as well as creatinine (6).

Notably, while foundational research exists on hyperuricemia among high-altitude indigenous populations, the landscape of hyperuricemia in high-altitude migrant populations remains poorly characterized. In-depth exploration of hyperuricemia-related factors in high-altitude migrants is thus of critical importance. This study utilizes physical examination data from high-altitude migrants at Ritu County Hospital (June–December 2024) to analyze hyperuricemia-related factors in this population.

## 2 Materials and methods

### 2.1 Research subjects

The population who underwent physical examinations at Ritu County Hospital from June to December 2024 were selected as the research subjects. All subjects were migrants who moved from low-altitude areas to work on the plateau. As the average altitude of Ritu County exceeds 4,000 meters and the annual outdoor working period is approximately 8–10 months, it was difficult to collect data on plateau residence durations exceeding 1 year. Therefore, the study population was defined as individuals with a plateau residence duration of 6 months or more.

### 2.2 Inclusion and exclusion criteria

Inclusion and exclusion criteria were strictly applied to ensure the homogeneity of the study population: (1) age > 18 years; (2) migration from low-altitude areas (altitude < 1,500 m) to high-altitude regions with ≥6 months of continuous residence; (3) no use of uric acid-regulating drugs (e.g., allopurinol, febuxostat) within the prior 3 months.

### 2.3 Research variables

General demographic information (including gender, age), blood routine indicators (white blood cell count, lymphocyte count, red blood cell count, etc.), biochemical markers (aspartate aminotransferase, alanine aminotransferase, total protein, albumin, etc.), and other relevant parameters (such as creatinine, urea, triglyceride, etc.) of the plateau migrant subjects were collected.

### 2.4 Research methods

Univariate analysis: For continuous variables, the t-test was used to compare the differences between the hyperuricemia group and the normal group. For categorical variables, the chi-square test was applied to analyze their correlation with hyperuricemia in high-altitude area migrants. A p-value less than 0.05 was considered statistically significant.

Multicollinearity analysis: Multicollinearity can lead to inaccurate model estimations, such as an increase in the standard error of coefficient estimation and the coefficient sign not conforming to the actual situation. Removing such variables helps improve the stability and interpretability of the model. The variance inflation factor (VIF) of each variable was calculated. Variables with a VIF greater than 5 were regarded as having serious collinearity and were excluded from subsequent analyses.

Multivariate logistic regression analysis: The remaining variables after multicollinearity analysis were included in the multivariate logistic regression model, with hyperuricemia as the dependent variable (yes = 1, no = 0). This was done to determine the factors independently related to hyperuricemia in plateau migrants and calculate the odds ratio (OR) and its 95% confidence interval (CI).

## 3 Results

### 3.1 Characteristics statistics of participants

**Figure 1:**
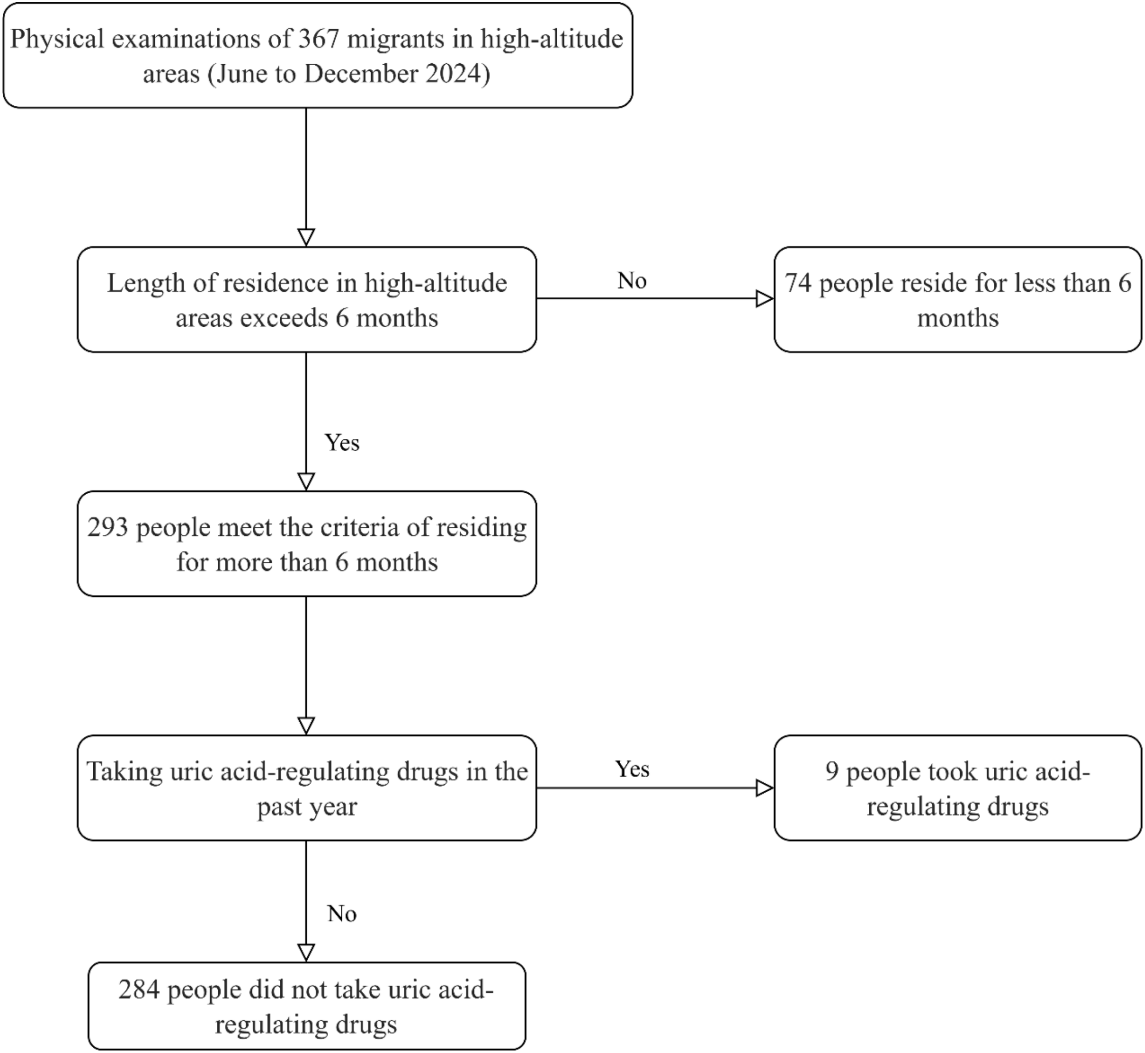
Flow chart for screening research population.

The screening process is as follows: A total of 367 plateau migrants who came for physical examinations from June to December 2024 were initially included. First, 74 individuals who had resided in high-altitude areas for less than 6 months were excluded, leaving 293 people who met the requirement of a residence duration of 6 months or more. Subsequently, 9 individuals who had taken uric acid-regulating drugs in the past year were excluded, resulting in a final inclusion of 284 plateau migrant participants who had not taken uric acid-regulating drugs. Among them, 224 were diagnosed with hyperuricemia, and 60 had normal uric acid levels. Specific screening process is shown in **Figure 1**.

A comprehensive and detailed comparative analysis was conducted on the various indicators of these plateau migrants. The detailed data of this comparative analysis are presented in **Table 1** below. In terms of gender distribution, the proportion of males in the hyperuricemia population was significantly higher than that in the normal population. The average age of the normal population was 29.62 ± 6.94 years old, while the average age of the hyperuricemia population was 27.36 ± 5.31 years old. The calculated t-value was 2.34, and the corresponding p-value was 0.02 (p < 0.05), indicating a significant age difference between the two groups, with the hyperuricemia population having a relatively lower average age. Further analysis revealed extremely significant differences between the two groups in key indicators such as red blood cell count, hemoglobin, urea, creatinine, and triglyceride.

**Table 1:** Characteristics statistics of participants.

Variables	Uric Acid (UA) (X ± SD)		X^2^/t	p
	Normal (n=60)	Hyperuricemia (n=224)		
Gender			36.87	<0.01
Female	12	2		
Male	48	222		
Age	29.62 ± 6.94	27.36 ± 5.31	2.34	0.02
White Blood Cell Count	7.28 ± 3.20	6.90 ± 2.30	0.87	0.39
Lymphocyte Count	2.07 ± 0.84	2.21 ± 0.93	-1.11	0.27
Intermediate Cell Count	0.47 ± 0.18	0.47 ± 0.30	-0.09	0.92
Neutrophil Count	4.75 ± 3.11	4.21 ± 1.95	1.26	0.21
Lymphocyte Percentage	31.38 ± 11.37	33.20 ± 9.72	-1.24	0.22
Intermediate Cell Percentage	6.99 ± 1.80	7.02 ± 2.24	-0.12	0.9
Neutrophil Percentage	61.63 ± 12.03	59.78 ± 10.12	1.21	0.23
Red Blood Cell Count	5.72 ± 0.51	6.11 ± 0.51	-5.24	<0.01
Hemoglobin	180.70 ± 19.17	193.95 ± 17.97	-5	<0.01
Mean Corpuscular Hemoglobin Concentration	345.18 ± 9.64	348.24 ± 9.09	-2.28	0.02
Mean Corpuscular Volume	91.47 ± 5.60	91.25 ± 6.22	0.26	0.8
Mean Corpuscular Hemoglobin	31.56 ± 2.39	31.77 ± 2.54	-0.57	0.57
Red Cell Distribution Width Coefficient of Variation	13.83 ± 0.94	13.75 ± 1.12	0.47	0.64
Hematocrit	52.20 ± 4.68	55.60 ± 4.82	-4.88	<0.01
Platelet Count	250.93 ± 56.69	245.06 ± 50.50	0.73	0.47
Mean Platelet Volume	8.23 ± 0.75	8.35 ± 0.72	-1.13	0.26
Platelet Distribution Width	16.17 ± 0.27	16.20 ± 0.27	-0.66	0.51
Platelet Hematocrit	0.20 ± 0.04	0.20 ± 0.04	0.23	0.82
Large Platelet Ratio	25.01 ± 5.70	25.81 ± 5.55	-0.98	0.33
Red Cell Distribution Width Standard Deviation	45.91 ± 3.15	45.62 ± 3.25	0.61	0.54
Aspartate Aminotransferase	17.38 ± 7.42	20.43 ± 19.46	-1.19	0.24
Alanine Aminotransferase	21.07 ± 16.50	24.83 ± 20.04	-1.34	0.18
AST/ALT	0.98 ± 0.32	0.97 ± 0.46	0.14	0.89
Total Protein	71.73 ± 4.63	73.73 ± 4.48	-3.04	<0.01
Albumin	40.78 ± 2.80	42.02 ± 2.20	-3.63	<0.01
Globulin	30.95 ± 3.12	31.71 ± 3.35	-1.58	0.11
A/G	1.32 ± 0.14	1.33 ± 0.14	-0.43	0.66
Total Bilirubin	15.85 ± 7.10	20.41 ± 10.79	-3.91	<0.01
Direct Bilirubin	3.85 ± 1.58	4.46 ± 2.45	-2.34	0.02
Indirect Bilirubin	12.00 ± 6.18	15.95 ± 8.98	-3.95	<0.01
Alkaline Phosphatase	58.55 ± 18.56	63.46 ± 16.65	-1.98	0.05
γ-Glutamyl transferase	26.43 ± 22.96	28.13 ± 19.99	-0.57	0.57
Urea	5.04 ± 1.05	5.59 ± 1.12	-3.43	<0.01
Creatinine	65.97 ± 13.71	74.23 ± 11.60	-4.71	<0.01
Urea/Creatinine	0.07 ± 0.02	0.07 ± 0.02	0.5	0.62
Creatine Kinase	88.38 ± 53.68	117.85 ± 140.23	-1.59	0.11
Creatine Kinase MB	16.25 ± 5.99	21.26 ± 28.62	-1.35	0.18
Lactate Dehydrogenase	135.63 ± 38.24	154.74 ± 82.69	-1.74	0.08
Triglyceride	1.64 ± 0.67	2.06 ± 1.52	-3.13	<0.01
Total Cholesterol	3.74 ± 0.70	3.97 ± 0.82	-1.96	0.05
High - Density Lipoprotein Cholesterol	1.41 ± 0.45	1.45 ± 0.36	-0.77	0.44
Low - Density Lipoprotein Cholesterol	1.90 ± 0.47	2.01 ± 0.55	-1.41	0.16
Calcium	2.03 ± 0.15	2.24 ± 0.18	-7.89	<0.01
Magnesium	0.92 ± 0.09	0.94 ± 0.09	-1.7	0.09
Carbon Dioxide	20.77 ± 3.26	20.56 ± 3.31	0.43	0.66
Glucose	5.98 ± 0.84	6.04 ± 0.81	-0.49	0.62
Amylase	64.63 ± 90.75	53.71 ± 19.90	0.93	0.36
Rheumatoid Factor	10.38 ± 9.73	9.80 ± 13.44	0.31	0.75
Lactate	21.18 ± 6.99	22.45 ± 8.52	-1.06	0.29
Total Bile Acid	1.89 ± 2.19	1.98 ± 3.10	-0.21	0.83

### 3.2 Multicollinearity analysis

To ensure that the multiple regression model was not affected by collinearity issues, a multicollinearity analysis was performed on the independent variables that were significantly related to hyperuricemia in the univariate analysis of plateau migrants. The results of the multicollinearity analysis are shown in **Table 2** as follows. The results showed that 6 variables, namely hemoglobin, mean cell hemoglobin concentration, hematocrit, total bilirubin, direct bilirubin, and indirect bilirubin, had a VIF greater than 5, indicating serious collinearity. These variables were removed from subsequent analyses.

**Table 2:** Multicollinearity analysis.

Collinearity diagnosis	VIF value	Tolerance
Gender	1.22	0.82
Age	1.14	0.88
Red Blood Cell Count	2.36	0.42
Hemoglobin	1507.63	0
Mean Corpuscular Hemoglobin Concentration	100.9	0.01
Hematocrit	1229.43	0
Total Protein	2.44	0.41
Albumin	3.13	0.32
Total Bilirubin	1.7977E+15	0
Direct Bilirubin	1.2378E+14	0
Indirect Bilirubin	1.7145E+15	0
Alkaline Phosphatase	1.08	0.92
Urea	1.17	0.85
Creatinine	1.3	0.77
Triglyceride	1.19	0.84
Calcium	1.86	0.54

### 3.3 Results of multivariate logistic regression analysis

The variables finally included in the model and the results are presented in **Table 3**. The results indicate that variables such as gender, age, red blood cell count, and creatinine are independently related to hyperuricemia in plateau migrants. The risk of men developing hyperuricemia is 6.73 times that of women. For every 1-year increase in age, the risk of developing hyperuricemia decreases by about 7%. For every 1-unit increase in red blood cell count, the risk of developing hyperuricemia increases by about 2.51 times. For every 1-unit increase in creatinine, the risk of developing hyperuricemia increases by about 4%.

**Table 3:** Results of multivariate logistic regression analysis.

Variables	b	SE	z	Wald χ2	p	OR	OR (95% CI)
Gender	1.60	0.37	4.35	18.91	<0.01	4.97	2.41 ~ 10.24
Age	-0.07	0.03	-2.26	5.10	0.02	0.94	0.88 ~ 0.99
Red Blood Cell Count	1.29	0.42	3.05	9.32	<0.01	3.63	1.57 ~ 8.31
Total Protein	0.05	0.06	0.90	0.80	0.37	1.05	0.94 ~ 1.18
Albumin	0.07	0.11	0.65	0.42	0.52	1.07	0.87 ~ 1.32
Alkaline Phosphatase	0.01	0.01	1.16	1.33	0.25	1.01	0.99 ~ 1.03
Urea	0.19	0.18	1.07	1.14	0.29	1.21	0.86 ~ 1.70
Creatinine	0.03	0.02	2.24	5.00	0.03	1.03	1.01 ~ 1.07
Triglyceride	0.38	0.20	1.91	3.64	0.06	1.47	0.99 ~ 2.17
Calcium	1.10	1.47	0.75	0.56	0.45	3.00	0.17 ~ 52.99

## 4 Discussion

This study demonstrated that factors such as gender, age, red blood cell count, and creatinine are independently correlated with hyperuricemia in high-altitude area migrants, providing crucial insights into the pathogenesis of hyperuricemia in this specific population.

The incidence rate of hyperuricemia in plateau migrants is as high as 78.87%, which is much higher than that in plain areas (14) and is the result of the joint action of multiple factors. Among the environmental factors, the hypoxia condition is particularly crucial. The hypoxic environment in high-altitude regions has a profound impact on the human metabolic system of migrants. On the one hand, continuous hypoxia prompts the body to initiate a series of stress responses in order to ensure the oxygen supply to vital organs (15). Among them, hypoxia-inducible factors are activated in large quantities. It not only further stimulates the excessive secretion of erythropoietin (16), triggering a sharp proliferation of red blood cells in plateau migrants (17), leading to a significant increase in blood viscosity and a remarkable slowdown in blood flow velocity (18). Several studies have shown that long-term exposure to high altitudes (ranging from 2,800 to 5,800 meters) can increase hematocrit levels in migrants (19–22). This increase is accompanied by an elevation in hemoglobin levels and blood viscosity. Due to the increase in hematocrit, renal plasma flow is reduced (23, 24). Under such a low-perfusion state, the uric acid excretion pathway of the kidneys is blocked, and the excretion capacity is severely restricted (25). On the other hand, hypoxia can also directly interfere with the intracellular energy metabolism pathway, forcing cells to rely more on anaerobic glycolysis for energy supply (26). During this process, the *de novo* synthesis pathway of purine nucleotides is abnormally activated (27), generating a large number of purines continuously, and the uric acid produced by subsequent metabolism also increases dramatically (28).

The incidence of hyperuricemia in men is much higher than that in women in high-altitude area migrants, which is mainly attributed to multiple factors. At the physiological level, men have higher androgen levels, while estrogen in women helps excrete uric acid (29). Notably, testosterone can stimulate the activity of isolated xanthine oxidase and increase circulating uric acid levels (30), further explaining the gender disparity in hyperuricemia prevalence. In terms of lifestyle, men among plateau migrants are more likely to drink alcohol to cope with the cold in high-altitude areas. Alcohol can impede uric acid excretion and compete with uric acid for excretion channels (31). At the same time, they consume more high-purine foods. In addition, due to differences in environmental adaptation, the oxygen is thin in high-altitude areas. Men’s stronger erythropoiesis ability leads to more uric acid precursors produced by red blood cell metabolism (32). Moreover, men have higher muscle mass, and the metabolic process also contributes to increased uric acid production (33).

In high-altitude area migrants, the incidence of hyperuricemia among young people is relatively high, which is the result of the combined action of multiple factors. Young individuals generally consume more high-purine foods than their older counterparts. Such dietary patterns directly lead to increased uric acid production (34). At the environmental adaptation level, the hypoxic environment at high altitudes prompts the young people’s bodies to produce more red blood cells (35, 36), whose metabolism generates more uric acid precursors.

The association between red blood cell count and hyperuricemia is likely closely related to the special environment in high altitude areas for migrants. Stimulated by the low oxygen environment at high altitudes, the body increases red blood cell production to meet the oxygen demand. In the process of circulation, the increased number of red blood cells will have more cellular metabolic activities. Red blood cells contain a large amount of phosphoribosyl pyrophosphate, which is an important raw material for purine synthesis (37–39). During the metabolism of red blood cells, more purines will be produced. After metabolism, purines will generate uric acid, thus increasing the production of uric acid.

Creatinine is a key indicator reflecting renal function. It mainly comes from muscle metabolism and has a relatively stable production rate. It is filtered by the glomerulus and hardly reabsorbed by the renal tubules, so it can accurately reflect the filtration function of the kidneys. Once the level of creatinine increases, it indicates that the glomerular filtration rate of the kidneys has declined and the ability of the kidneys to remove metabolic wastes has weakened (40, 41). As a result, creatinine accumulates in the body and its level in the blood rises. The excretion of uric acid highly depends on the kidneys. The dynamic balance of blood uric acid is maintained through glomerular filtration, renal tubular reabsorption and secretion. When the renal function is impaired and the filtration rate decreases, the excretion pathway of uric acid will be blocked. Unable to be excreted normally, uric acid will accumulate continuously in the blood, leading to an increase in the content of uric acid in the blood (42).

However, this study has several notable limitations. First, the research data are solely derived from the physical examination population at Ritu County Hospital, potentially leading to selection bias as the sample only includes high-altitude migrants and excludes indigenous populations, making it impossible to compare hyperuricemia characteristics or risk factors between migrant and indigenous groups. This limitation restricts the ability to distinguish whether hyperuricemia in migrants is driven by environmental adaptation or genetic/epigenetic factors inherent in indigenous populations, thus limiting the understanding of disease heterogeneity in high-altitude regions. Second, key clinical variables were underrepresented, including detailed migration duration, body mass index (BMI), smoking status, and oxygen uptake levels. The absence of these variables may obscure potential confounding effects or dose-response relationships. In our study, as the included population was plateau migrant workers, females accounted for a relatively small proportion in physical labor at high altitudes. This resulted in a low proportion of females in the study (only 2 females in the hyperuricemia group and 12 in the normal group), which may limit the conclusion that gender is a related factor for hyperuricemia. The small sample size of females may overestimate the association between male gender and hyperuricemia, and insufficient female data hinder accurate evaluation of estrogen’s protective effect and gender-specific risks, leading to male-biased conclusions with limited generalizability to females.

## 5 Conclusion

This study investigated the related factors of hyperuricemia in high-altitude area migrants using the physical examination population at Ritu County Hospital as the research object. The results showed that the incidence of hyperuricemia in this migrant population was 78.87%. Through a series of analyses, gender, age, red blood cell count, and creatinine were identified as independently related to hyperuricemia in plateau migrants. Men had a higher risk of developing the disease, the risk decreased with age, and an increase in red blood cell count and creatinine increased the risk of developing the disease. These findings are of great significance for both clinical practice and public health among high-altitude area migrants. Clinically, they assist doctors in early diagnosis and treatment of key populations and the formulation of personalized treatment plans for migrants.

## Data Availability

The datasets generated during the current study are available from the corresponding author upon reasonable request.
